# Assessment of serum macrophage migration inhibitory factor (MIF), adiponectin, and other adipokines as potential markers of proteinuria and renal dysfunction in lupus nephritis: a cross-sectional study

**DOI:** 10.1186/s40364-020-00236-x

**Published:** 2020-10-28

**Authors:** Jorge Ivan Gamez-Nava, Valeria Diaz-Rizo, Edsaul Emilio Perez-Guerrero, Jose Francisco Muñoz-Valle, Ana Miriam Saldaña-Cruz, Nicte Selene Fajardo-Robledo, Heriberto Jacobo-Cuevas, Cesar Arturo Nava-Valdivia, Miriam Fabiola Alcaraz-Lopez, Xochitl Trujillo, Miguel Huerta, Ernesto German Cardona-Muñoz, Laura Gonzalez-Lopez

**Affiliations:** 1 Programa de Doctorado en Farmacología del Departamento de Fisiología y Programa de Doctorado en Salud del Pública Depatamento de Salud Pública, Universidad de Guadalajara, Centro Universitario de Ciencias de la Salud, Sierra Mojada 950, Colonia Independencia, 44340 Guadalajara, Jalisco Mexico; 2 Centro Medico Nacional de Occidente, Unidad de Investigacion Biomedica 02, Instituto Mexicano del Seguro Social, Hospital de Especialidades, 44340 Guadalajara, Jalisco Mexico; 3 Departamento de Disciplinas Filosófico, Metodológico e Instrumentales, Universidad de Guadalajara, Centro Universitario de Ciencias de la Salud, 44340 Guadalajara, Jalisco Mexico; 4 Universidad de Guadalajara, Centro Universitario de Ciencias de la Salud, Instituto de Investigación en Ciencias Biomédicas, 44340 Guadalajara, Jalisco Mexico; 5 Departamento de Fisiología, Universidad de Guadalajara, Centro Universitario de Ciencias de la Salud, Sierra Mojada 950, Colonia Independencia, 44340 Guadalajara, Jalisco Mexico; 6 Laboratorio de Investigación y Desarrollo Farmacéutico, Universidad de Guadalajara, Centro Universitario de Ciencias Exactas e Ingenierías, 44430 Guadalajara, Mexico; 7 Departamento de Microbiología y Patología, Universidad de Guadalajara, Centro Universitario de Ciencias de la Salud, 44340 Guadalajara, Jalisco Mexico; 8 Instituto Mexicano del Seguro Social, HGR45, 44340 Guadalajara, Jalisco Mexico; 9 Universidad de Colima, Centro Universitario de Investigaciones Biomédicas, 28040 Colima, Mexico; 10 Departamento de Medicina InternaReumatología, Instituto Mexicano del Seguro Social (IMSS), Hospital General Regional 110, 44716 Guadalajara, Jalisco Mexico

**Keywords:** Lupus nephritis, Lupus Erythematosus systemic, MIF, Adiponectin, Adipokines, Biomarkers

## Abstract

**Background:**

To date, the association of serum macrophage migration inhibitory factor (MIF) and serum adipokines with lupus nephritis is controversial.

**Objective:**

To assess the utility of serum MIF, leptin, adiponectin and resistin levels as markers of proteinuria and renal dysfunction in lupus nephritis.

**Methods:**

Cross-sectional study including 196 systemic lupus erythematosus (SLE) patients and 52 healthy controls (HCs). Disease activity was assessed by Systemic Lupus Erythematosus Disease Activity Index (SLEDAI). Renal SLE involvement was investigated by renal-SLEDAI. MIF, adiponectin, leptin and resistin levels were quantified by ELISA. We assessed the correlations of quantitative variables by Spearman correlation (r_s_). Multivariable linear regression adjusted the variables associated with the severity of proteinuria.

**Results:**

SLE patients had higher MIF (*p* = 0.02) and adiponectin (*p* < 0.001) than HCs. Patients with renal SLE involvement (*n* = 43) had higher adiponectin (19.0 vs 13.3 μg/mL, *p* = 0.002) and resistin (10.7 vs 8.9 ng/mL, *p* = 0.01) than patients with non-renal SLE (*n* = 153). Proteinuria correlated with high adiponectin (*r*_*s*_ = 0.19, *p* < 0.009) and resistin (*r*_*s*_ = 0.26, *p* < 0.001). MIF (*r*_*s*_ = 0.27, *p* = 0.04). Resistin correlated with increased creatinine (*r*_*s*_ = 0.18, *p* = 0.02). High renal-SLEDAI correlated with adiponectin (*r*_*s*_ = 0.21, *p* = 0.004). Multiple linear regression showed that elevated adiponectin (*p* = 0.02), younger age (*p* = 0.04) and low MIF (*p* = 0.02) were associated with the severity of proteinuria. Low MIF and high adiponectin levels interacted to explain the association with the severity of proteinuria (R^2^ = 0.41).

**Conclusions:**

High adiponectin combined with low MIF concentrations int+eract to explain the severity of proteinuria in renal SLE. These findings highlight the relevance of adiponectin, resistin and MIF as markers of LN.

## Background

Systemic lupus erythematosus (SLE) is considered a chronic inflammatory autoimmune disorder characterised by an extensive spectrum of inflammation in organs and tissues, with the kidneys being one of the main organs affected by the disease. The cumulative incidence of renal involvement in SLE is 54%, and it has a prevalence that varies from 30 to 80% [1, 2]. Lupus nephritis (LN) is associated with significant morbidity and mortality, with an incidence of end-stage renal disease (ESRD) of 27.6 per 1000 patient-years, although this pattern can differ depending on race [3]. For instance, Hispanic patients have a high predisposition to LN, similar to that of Asian and African American populations and higher than that of Caucasians [4]. One of the main strategies to detect patients at risk of LN is using clinical markers. Traditional markers of LN, such as increased native double-stranded DNA antibody (anti-dsDNA) and decreased C3 and C4 complement fractions, are currently used in the clinical assessment of LN [5]; however, these markers do not appear to have sufficient sensitivity for detecting LN in a subgroup of SLE patients, thus, new non-traditional markers should be tested [6]. Among these other markers of LN are included the serum levels of adipokines and cytokines. These molecules have been associated with the parameters of renal involvement, such as proteinuria, decrease in glomerular filtration rate, impairment of creatinine clearance, haematuria, and increase in urinary leucocytes or casts [7–10]**.** Nevertheless, some cytokines and adipokines such as the serum levels of the macrophage migration inhibitory factor (MIF), leptin, adiponectin and resistin have been insufficiently evaluated as markers in LN. MIF is a pleiotropic cytokine with a wide variety of proinflammatory and immunomodulatory functions, including the regulation of the inflammatory response mediated by T-cells [11, 12]. Although MIF is constitutively expressed in many tissues, in the kidneys, it can be expressed mainly by glomerular epithelial cells and cortical tubules [13]. Experimental models of glomerulonephritis have shown an upregulation of renal mRNA MIF, leading to the overexpression of MIF [14]. In models of kidney damage, the overexpression of MIF in podocytes induces progressive injury to these cells and progression to glomerulosclerosis [15]. In SLE patients, the participation of MIF in the disease activity of SLE has been investigated by a few studies. Tu et al. found an increase in the serum MIF levels in SLE patients compared with controls, and MIF levels correlated with the severity of disease activity measured by the Systemic Lupus Erythematosus Disease Activity Index (SLEDAI) [16]. Moreover, to date the relevance of MIF in LN has been infrequently evaluated, assessed mainly in relationship with MIF urinary concentrations [17, 18]. Some authors have examined the association between urinary MIF levels and proteinuria. Brown et al. did not observe a relationship between serum or urinary MIF levels and the amount of proteinuria [17]. However, these authors identified an increase in urinary MIF only in patients with proliferative nephritis [17]. On the other hand, Vincent et al. did not identify correlations between urinary MIF and nephritis in SLE patients, although high concentrations of urinary MIF were related to high disease activity in these patients [18]. In the clinical context, the presence and severity of proteinuria is a key variable to suspect LN, and it has been included as one of the items described in the renal-SLEDAI to identify renal disease activity [19]. Proteinuria is not only a clinical marker for LN but also recognised as a major prognostic factor of ESRD [20]. Therefore, the correlation between serum MIF levels and proteinuria requires a more detailed assessment.

Nowadays, there is some evidence supporting that MIF expression can be related with certain adipokines. For instance, Koska et al. observed that the mRNA expression of MIF in adipocytes is negatively associated with the levels of adiponectin [21]. However, currently, there is a lack of information regarding the relation between serum MIF levels and adipokines in LN, and it has not been determined whether the association observed between adipokines and renal flare in SLE is independent of MIF levels.

In LN, some adipokines, such as leptin, resistin and adiponectin, have been investigated with discordant results. For instance, Hutcheson et al. found that adiponectin concentrations decrease with the increase in disease activity [8], whereas our group observed a positive correlation between high adiponectin levels and the severity of proteinuria [11].

In consequence, there are insufficient data regarding the possible role of MIF and adipokines levels as markers of renal involvement in SLE patients. Therefore, we decided to assess the value of serum MIF, leptin, adiponectin and resistin levels as markers of proteinuria and renal dysfunction in LN.

## Methods

### Study design

We conducted a cross-sectional study of female patients with SLE. These patients met the following inclusion criteria: a) a diagnosis of SLE as corroborated by a rheumatologist; b) met the 1982 American College of Rheumatology criteria of SLE [22]; c) age ≥ 18 years; d) ethnicity of Mexican-Mestizos [23]; and e) disease duration of at least 1 year since the first symptom. We excluded patients with overlap syndrome, pregnancy, active infection, and diseases other than SLE that might produce abnormal proteinuria.

### Clinical setting

All patients were selected from the lupus cohort of an outpatient rheumatology clinic of one largest secondary-care centres in Guadalajara, Mexico (Hospital General Regional 110, Instituto Mexicano del Seguro Social [IMSS]).

A healthy controls (HCs) group of 52 females matched by age and ethnicity were selected. These controls were clinically healthy subjects recruited from patients visiting the Department of Preventive Medicine for check-ups at the same hospital. HCs were included to compare the values of MIF and adipokines with those of the SLE patients.

### Clinical evaluations

Three trained rheumatologist researchers assessed the SLE patients using a structured chart, including disease features, comorbid diseases, and current pharmacological treatment. Assessment of disease damage: This was investigated using the Systemic Lupus International Collaborating Clinics/American College of Rheumatology (SLICC/ACR) damage index [24]. Assessment of disease activity: This assessment was performed using the Systemic Lupus Erythematosus Disease Activity Index (SLEDAI) [19]. The SLEDAI is an index designed to assess disease activity in the preceding 10 days, with 24 weighted clinical and laboratory variables corresponding to 9 different organs/systems. The SLEDAI score ranges from 0 to 105. Renal activity was evaluated with the renal-SLEDAI (rSLEDAI) [19], which represents the sum of the renal items of the SLEDAI. The rSLEDAI includes the following items: proteinuria, pyuria, erythrocyturia, and urine casts; each one is scored with 0 meaning absence or 4 points meaning presence; therefore, the maximum rSLEDAI is 16 [19]. The main criterion of renal activity was proteinuria greater than 0.5 g/day or in conjunction with any of the following features: persistent haematuria, leucocytes in urine or urine casts by granulocytes or erythrocytes (excluding other causes). SLE patients with these features were classified as the renal SLE group, and this group was compared with the non-renal group, which consisted of SLE patients that did not meet any criteria of the rSLEDAI. We used the Mexican version of the SLEDAI (MEX-SLEDAI) [25]. The MEX-SLEDAI is an adaptation of the SLEDAI that does not include laboratory immunological parameters such as complement fraction and anti-dsDNA [25]. The MEX-SLEDAI score ranges from 0 to 32.

### MIF and adipokine measurements

Blood samples were extracted from SLE patients and HCs at the time of clinical evaluation. Samples were stored at room temperature for 30 min after sampling. Then, the samples were centrifuged at 1300×g for 15 min at 4 °C. Serum samples were stored at − 80 °C without freeze-thaw cycles for a maximum of 6 months. All serum samples were coded before the measurements. This strategy was made to blind the researchers who assessed the clinical characteristics to the MIF and adipokine results, minimising the risk of measurement bias. Serum MIF levels were quantified using a commercial ELISA kit (R&D™, Minneapolis, USA). The sensitivity of this assay was 0.068 ng/mL. Measurements of adipokines, including leptin (sensitivity of 7.8 pg/mL), adiponectin (sensitivity of 0.89 ng/mL), and resistin (sensitivity of 0.055 ng/mL), were performed using commercial ELISA kits (R&D™, Minneapolis, USA). If required, we performed a serum sample dilution in cases with values above the highest point on the standard curve. All ELISAs were performed according to the manufacturer’s instructions. All samples were run in duplicate to improve assay precision.

### Statistical analysis

Quantitative variables are described as medians (ranges), and qualitative characteristics are described as frequencies (%). The chi-square test (or Fisher’s exact test) was used for comparisons between proportions. Comparisons of quantitative variables between the renal-SLE and non-renal groups were performed using the Mann-Whitney U test. To compare the differences in quantitative variables among the three groups (renal SLE, non-renal SLE, and HCs), we used the Kruskal-Wallis test. In this analysis, *p-values* for multiple comparisons were adjusted by Bonferroni correction. To identify the correlations between MIF, adipokines and other clinical variables, Spearman’s test was performed. Multiple regression analysis was used (stepwise method) to identify variables associated with proteinuria (g/day). In this model, variables with biological plausibility and statistical significance (*p* < 0.20) in univariate analysis were introduced as covariables.

We further explored the possible interaction effect of MIF and adipokines. For this proposed interaction, we constructed individual regression models. Before the construction of interaction models, we first evaluated the collinearity between MIF and adipokines and their product term. We used the mean centring method for testing interactions as described below [26, 27]. In brief, for each of the tested predictor variables, the mean was subtracted before testing the products to represent their interaction. Then, models of the interactions of multiplicative terms were tested for each of the transformed variables (correcting for MIF and adipokines). We used SPSS Statistics for Windows (Version 25.0, IBM Corp., Armonk, NY) and R version 4.0.0 [28] to perform the statistical analyses. Figures were constructed in R using the ggplot2 package [29]. A *p-value* ≤ 0.05 was considered statistically significant.

## Results

### Comparison of healthy controls and SLE patients

This study included 196 SLE patients and 52 HCs. Table 1 shows comparisons of the clinical variables between the SLE and HCs groups. All SLE patients and HCs were females and Mexican-Mestizo. SLE patients had a similar median age compared with HCs (45 vs 47 years, *p* = 0.87). Body mass index (BMI) was not significantly different between the SLE and HCs groups (27.3 vs 27.9, *p* = 0.86).

**Table 1:** Comparison of clinical variables between SLE patients and healthy controls

Variable	SLE *n* = 196	Healthy Controls *n* = 52	p
Age (years) ^a^	45 (18–73)	47 (22–54)	0.87
Gender ^b^	196 (100)	52 (100)	–
Mexican-Mestizo ^b^	196 (100)	52 (100)	–
BMI (kg/m^2^) ^a^	27.3 (17.7–40.0)	27.9 (18.4–47.3)	0.86
SLE duration (years) ^a^	8.3 (2–28)	–	–
C3 fraction complement (mg/dL) ^a^	142.0 (42.0–252.0)	–	–
C4 fraction complement (mg/dL) ^a^	31 (6.6–71.7)	–	–
Positive anti-dsDNA ^b^	55 (28.1)	–	–
SLEDAI (score) ^a^	2 (0–12)	–	–
rSLEDAI (score) ^a^	0 (0–12)	–	–
- Renal-SLE patients ^b^	43 (21.9)	–	–
SLICC/ACR (score) ^a^	1 (0–5)	–	–
MEX-SLEDAI (score) ^a^	1 (0–10)	–	–
Glucocorticoids ^b^	196 (100)	–	–
- Prednisone > 10 mg/day ^b^	60 (30.6)	–	–
Immunosuppressive drugs ^b^	145 (74.0)	–	–
- Azathioprine users ^b^	91 (46.4)	–	–
- Cyclophosphamide users ^b^	13 (6.6)	–	–
- Mycophenolate users ^b^	56 (28.5)	–	–
Other drugs (Methotrexate) ^b^	27 (13.8)		

^a^ Data expressed as medians and ranges (minimum and maximum value). ^b^ Data provided in frequencies (percentages). *SLE* Systemic Lupus Erythematosus, *SLEDAI* original SLE Disease Activity Index, high score indicates higher disease activity, *SLICC/ACR* Systemic Lupus International Collaborating Clinics/American College of Rheumatology, *rSLEDAI* Renal-SLEDAI score (includes proteinuria greater than 0.5 g in 24 h, persistent hematuria, leucocytes on urine or urine casts -granulocytes or erythrocytes-), higher score indicates high renal disease activity. MEX-SLEDAI: Version of SLEDAI validated in Mexico. Comparisons between proportions: Chi- square (or Fisher exact test if required). Comparisons between quantitative variables: Mann-Whitney U test

### Characteristics of the SLE patients

Table 1 also includes a description of the selected characteristics of the total group of SLE patients. Of 196 SLE patients, 28.1% had positive anti-dsDNA antibodies. The median SLEDAI score was 2 points. Seventy-six (38.8%) SLE patients had active disease (SLEDAI> 4). The r-SLEDAI ranged from 0 to 12 points. Forty-three SLE patients (21.9%) presented renal disease activity. All SLE patients were receiving glucocorticoids, although only 60 (30.6%) were receiving a dosage > 10 mg/day. Of the total SLE patients, 74% were receiving immunosuppressive therapy.

### Comparison of MIF and adipokine levels between SLE and HCs

**Fig. 1:**
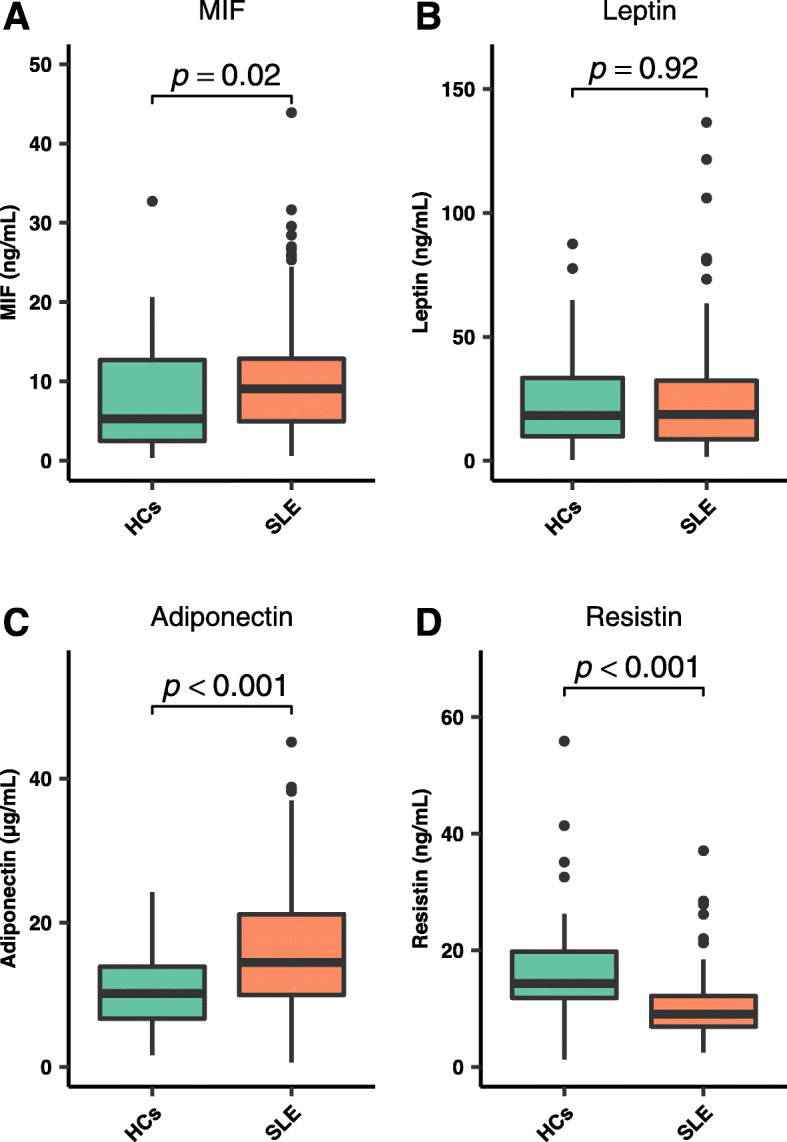
Comparison of MIF and adipokines between healthy controls (HCs) and systemic lupus erythematosus (SLE) patients. Comparisons were performed with the Mann-Whitney U test

Figure 1 presents the comparison of MIF and adipokine levels between the SLE and HCs groups. Serum MIF and adiponectin concentrations were higher in SLE patients than in HCs. Increased MIF levels were observed in SLE patients [9.1 (0.6–43.9) ng/mL vs. 5.3 (0.3–32.7) ng/mL, *p* = 0.02]. Adiponectin concentrations were also higher in SLE patients than in HCs [14.5 (0.6–45.1) μg/mL vs 10.2 (1.6–24.3) μg/mL, *p* < 0.001]. Resistin levels were lower in SLE patients than in HCs [9.1 (2.4–37.1) ng/mL vs 14.3 (1.3–55.9) ng/mL], *p* < 0.001). No differences were identified in the concentrations of leptin between SLE patients and HCs [18.6 (1.6–136.5) ng/mL vs 18.3 (0.31–87.48) ng/mL, *p* = 0.92].

### Comparison of MIF and adipokine levels in HCs, renal SLE and non-renal SLE

**Fig. 2:**
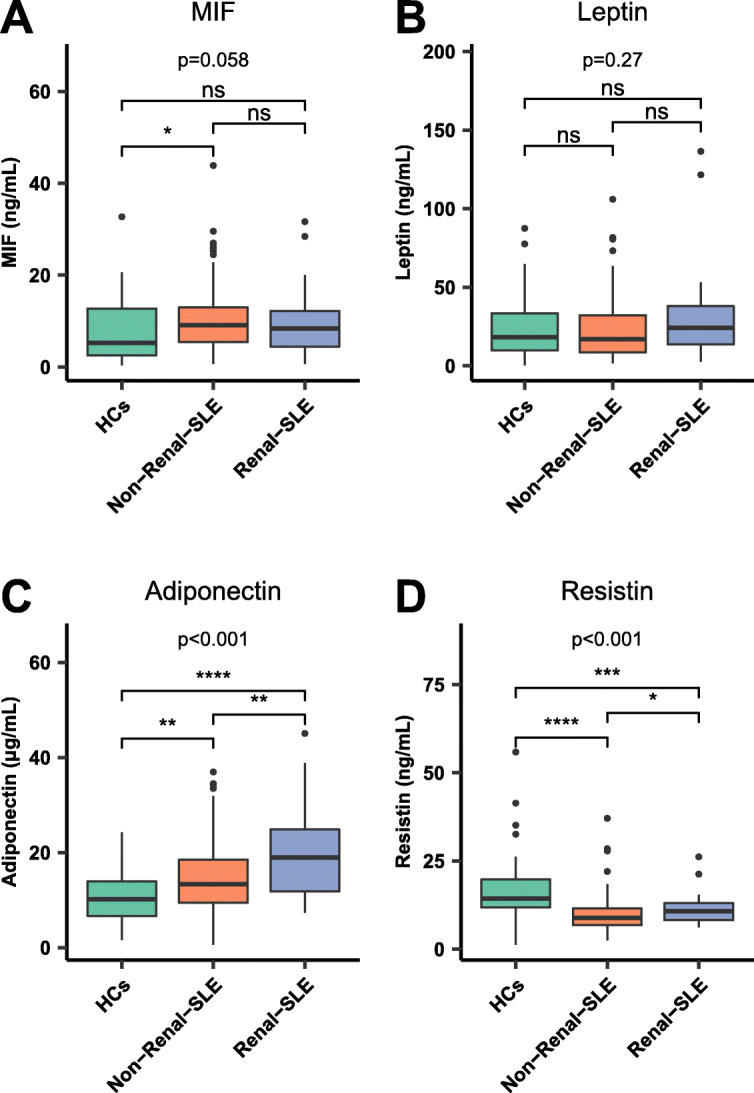
Comparison of MIF and adipokines in HCs, renal SLE, and non-renal SLE. HCs: healthy controls. SLE: Systemic lupus erythematosus. Renal SLE includes patients with proteinuria higher than 0.5 g/day as the sole criterion or in conjunction with persistent haematuria, leucocytes in urine or urine casts by granulocytes or erythrocytes. Comparisons between quantitative variables were performed with the Kruskal-Wallis test. *p* < 0.05. *P* values for multiple comparisons were adjusted by Bonferroni correction. ns: *p* > 0.05. *p < = 0.05, **p < = 0.01, ***p < = 0.001, ****p < = 0.0001

Figure 2 shows the comparison of the MIF and adipokine levels among the HCs, renal SLE and non-renal SLE groups. The three groups had differences in adiponectin levels (*p* < 0.001). Post hoc analysis showed that adiponectin concentrations were more elevated in renal SLE patients than in HCs [19.0 (7.3–45.1) μg/mL vs 10.2 (1.6–23.4) μg/mL, *p* < 0.001] and non-renal SLE patients [19.0 (7.3–45.1) μg/mL vs 13.3 (0.6–37.0) μg/mL, *p* = 0.002]. Non-renal SLE patients presented higher levels of adiponectin than HCs [13.3 (0.6–37.0) μg/mL vs 10.2 (1.6–23.4) μg/mL, *p* = 0.002]. Resistin concentrations were more elevated in HCs than in non-renal SLE patients [14.3 (1.3–55.9) ng/mL vs 8.9 (2.5–37.1) ng/mL, *p* < 0.001] and renal SLE patients [14.3 (1.3–55.9) ng/mL vs 10.7 (6.2–26.2) ng/mL, *p* < 0.001]. MIF and leptin levels were not significantly different among the three groups.

### Comparison of variables between renal SLE and non-renal SLE

Renal SLE patients received higher doses of prednisone than patients without renal activity [20 (2.5–75) mg/day vs 7.5 (2.5–50.0) mg/day, *p* < 0.001], but the frequency of the concurrent use of immunosuppressive drugs was similar in renal SLE and non-renal SLE patients (*p* = 0.64). Furthermore, renal SLE and non-renal SLE patients had similar disease durations [43 (18–62) years vs 46 (18–73) years, *p* = 0.13]. Other comparisons of variables between renal SLE patients and non-renal SLE patients are described in Table 2.

**Table 2:** Comparison of clinical variables between SLE patients without proteinuria and SLE patients with proteinuria

Variables	Non-Renal-SLE *n* = 153	Renal-SLE *n* = 43	p
Age (years) ^a^	46 (18–73)	43 (18–62)	0.13
Disease duration, (years) ^a^	9 (2–28)	6 (2–28)	0.10
C3 fraction complement (mg/dL) ^a^	142.0 (60.0–142.0)	154.5 (42.0–252.0)	0.84
C4 fraction complement (mg/dL) ^a^	31 (7.4–71.7)	31.3 (6.7–62.9)	0.92
Positive anti-dsDNA ^b^	39 (25.5)	16 (37.2)	0.27
SLEDAI (score) ^a^	2 (0–12)	6 (4–12)	< 0.001
Creatinine clearance (mL/min) ^a^	123.1 (96.6–158.3)	114.9 (80.8–147.0)	0.06
eGFR (mL/min/m2) ^a^	111.2 (29.6–258.9)	108.6 (17.1–182.9)	0.91
Serum creatinine (mg) ^a^	0.7 (0.6–2.2)	0.7 (0.4–3.7)	0.44
Immunosuppressive drugs ^b^	112 (73.2)	33 (76.7)	0.64
Glucocorticoid user ^b^	153 (100)	43 (100)	–
Glucocorticoid dose (mg/day) ^a^	7.5 (2.5–50.0)	20 (2.5–75.0)	< 0.001
Immunosuppressive drugs ^b^	112 (73.2)	33 (76.7)	0.69
- Azathioprine users ^b^	73 (47.7)	18 (41.8)	0.88
- Cyclophosphamide users ^b^	9 (5.9)	3 (9.3)	0.29
- Mycophenolate users ^b^	37 (24.2)	19 (44.2)	0.002
Others drugs (Methotrexate) ^b^	23 (15.0)	4 (9.3)	0.44

^a^ Data expressed as median and range (minimum and maximum value). ^b^ Data provided in percentages (n/total patients evaluated). *SLE* Systemic Lupus Erythematosus, *SLEDAI* SLE Disease Activity Index, *rSLEDAI* Renal SLEDAI, *MEX-SLEDAI* Mexican version of SLEDAI. Estimated glomerular filtration rate (eGFR). Renal-SLE includes patients with proteinuria greater than 0.5 g in 24 h, as sole criterion or in conjunction with persistent hematuria, leucocytes on urine or urine casts by granulocytes or erythrocytes. Glucocorticoids included: prednisone or deflazacort. GCs dose were expressed as equivalent to prednisone. Comparisons between proportions were compared with Chi- square or Fisher exact test (when required). Comparisons between quantitative variables: Mann-Whitney U test

### Correlations of MIF and adipokines with clinical variables

Table 3 describes the correlations of MIF and adipokines with clinical and laboratory variables. Lower serum MIF levels were correlated with increased age (*p* = 0.003) and longer duration of SLE (*p* = 0.004). MIF did not correlate with SLEDAI, rSLEDAI, proteinuria or other features. Serum leptin levels correlated with BMI (*p* < 0.001), proteinuria (*p* = 0.01) and estimated glomerular filtration rate (eGFR) (*p* = 0.02). High concentrations of adiponectin correlated with proteinuria (*p* = 0.009), rSLEDAI (*p* = 0.004), and high Mex-SLEDAI scores (*p* = 0.03). However, adiponectin levels were negatively correlated with eGFR (*p* = 0.05). Additionally, serum adiponectin levels correlated with glucocorticoid dose (*p* = 0.02) and BMI (*p* < 0.001). Correlations were observed between resistin levels and proteinuria (*p* < 0.001), serum creatinine (*p* = 0.02), SLICC/ACR (*p* = 0.01), and glucocorticoid dose (*p* = 0.03). No correlations were observed between serum leptin or resistin and proteinuria, SLEDAI or rSLEDAI.

**Table 3:** Correlations between cytokines and adipokines with clinical variables: including disease activity index, rSLEDAI score, individual markers of renal activity or renal dysfunction and glucocorticoids doses in SLE-patients

	MIF (ng/mL) *n* = 196		Leptin (ng/mL) *n* = 196		Adiponectin (μg/mL) *n* = 196		Resistin (ng/mL) *n* = 188	
	r_s_	p	r_s_	p	r_s_	p	r_s_	p
Age, years	**−0.21**	**0.003**	0.04	0.62	−0.13	0.07	0.03	0.67
BMI, kg/m^2^	− 0.06	0.36	**0.47**	**< 0.001**	**−0.27**	**< 0.001**	**0.21**	**0.004**
Disease duration, years	**−0.21**	**0.004**	0.06	0.38	−0.07	0.35	0.06	0.42
SLEDAI, score	0.04	0.54	0.09	0.22	0.11	0.13	0.03	0.70
rSLEDAI, score	−0.003	0.96	0.1	0.17	**0.21**	**0.004**	0.13	0.07
MEX-SLEDAI, score	0.02	0.75	0.1	0.13	**0.16**	**0.03**	0.04	0.58
SLICC/ACR, score	0.15	0.15	0.09	0.24	0.03	0.65	**0.19**	**0.01**
Proteinuria, g/24 h	−0.02	0.77	**0.18**	**0.01**	**0.19**	**0.009**	**0.26**	**< 0.001**
Creatinine, mg	**0.27**	**0.04**	0.12	0.1	0.16	0.22	**0.18**	**0.02**
eGFR (mL/min/m2)	0.11	0.13	**0.17**	**0.02**	**−0.15**	**0.05**	0.01	0.91
Creatinine clearance (mL/min)	0.07	0.39	0.1	0.89	−0.01	0.18	−0.06	0.44
GCs dose, mg/day	−0.04	0.63	0.1	0.15	**0.17**	**0.02**	**0.16**	**0.03**

*r*_*s*_ Spearman Rank Correlation, *BMI* Body mass index, *GCs* Glucocorticoids, *SLE* Systemic Lupus Erythematosus, *SLEDAI* SLE Disease Activity Index, *rSLEDAI* Renal SLEDAI, *MEX-SLEDAI* Mexican version of SLEDAI, *SLICC/ACR* Systemic Lupus International Collaborating Clinics/American College of Rheumatology, *eGFR* Estimated glomerular filtration rate. Glucocorticoids included: prednisone or deflazacort. GCs dose were expressed as equivalent to prednisone. Spearman Rank Correlation test *p* < 0.05

### Correlations of MIF and adipokines with parameters of renal activity in the 43 renal SLE patients

We investigated the correlations of MIF and adipokine levels with parameters of renal activity in renal SLE patients. MIF levels were significantly correlated with proteinuria in g/day (*r*_*s*_ = − 0.47; *p* = 0.008) but not with serum creatinine (*r*_*s*_ = − 0.24; *p* = 0.13), 24-h creatinine clearance (*r*_*s*_ = − 0.14; *p* = 0.40), or eGFR (*r*_*s*_ = 0.15; *p* = 0.33). Furthermore, in renal SLE patients, adiponectin levels correlated only with serum creatinine (*r*_*s*_ = 0.34; *p* = 0.03) but not with other parameters of renal activity. Resistin and leptin did not show any correlations with renal inflammatory features (data not shown).

### Variables associated with the severity of proteinuria (g/day): results of the multiple linear regression analysis

Table 4 demonstrates the findings of the factors associated with the severity of proteinuria in g/day obtained in the multiple linear regression analysis. With the enter method, the variables associated with the severity of proteinuria (g/day) were glucocorticoid dose (*p* < 0.001), adiponectin level (*p* < 0.001), MIF level (*p* = 0.01) and age (*p* < 0.001). Using the forward stepwise method in multivariable linear regression analysis, the factors associated with the severity of proteinuria in g/day were higher glucocorticoid doses (*p* < 0.001), higher adiponectin levels (*p* = 0.001), lower MIF levels (*p* = 0.005) and younger age (*p* = 0.011). This model was adjusted by glucocorticoid dose, immunosuppressive therapy, disease duration, age, and MIF, adiponectin, leptin, and resistin levels. The R^2^ and adjusted R^2^ of this model were 0.41 and 0.40, respectively.

**Table 4:** Variables associated with intensity of proteinuria in the linear regression analysis

	Proteinuria g/day			
	Univariable analysis		Multivariable analysis	
Independent Variables	β coefficient (IC 95%)	p	β coefficient (IC 95%)	p
GCs (mg/day)	0.07 (0.05 to 0.08)	< 0.001	0.05 (0.04 to 0.08)	< 0.001
Adiponectin levels (μg/mL)	0.09 (0.06 to 0.11)	< 0.001	0.05 (0.02 to 0.07)	0.001
MIF (ng/mL)	−0.04 (− 0.08 to − 0.01)	0.02	− 0.04 (− 0.07 to − 0.01)	0.005
Age (years)	−0.05 (− 0.07 to − 0.03)	< 0.001	−0.03 (− 0.05 to – 0.01)	0.011
Leptin, ng/mL	−0.00 (− 0.01 to 0.01)	0.70	Not significant to the model	
Resistin, ng/mL	0.03 (−0.02 to 0.09)	0.30	Not significant to the model	
Immunosuppressive drugs	–0.51 (-1.13 to 0.12)	0.12	Not significant to the model	

*Dependent variable*: quantity total of 24-h proteinuria. Multiple regression analysis was performed using stepwise method. Model was adjusted by disease duration, age, adiponectin, MIF, leptin, resistin, Glucocorticoid doses expressed as equivalent of prednisone doses (GCs) and using of immunosuppressive therapy. R^2^ for multivariable model was 0.41. Adjusted R^2^ for multivariable model was 0.40. Covariates included in this analysis were those variables with statistical significance in the univariate analysis or were considered with biological plausibility to proteinuria

We tested for interactions in the multiple regression analysis to assess weighted variables associated with the amount of proteinuria (data not shown). After testing for interactions, we identified an interaction between age and adiponectin levels as well as an interaction of serum MIF and adiponectin levels with the amount of proteinuria in SLE patients. In the interaction model, higher adiponectin levels and higher age increased proteinuria levels. Lower MIF levels interacted with higher adiponectin levels to increase proteinuria. The adjusted R^2^ of the final interaction model was 0.41 with *p* < 0.001.

## Discussion

In the bivariable analysis, an increase in the levels of the three adipokines (adiponectin, leptin and resistin) correlated with the severity of proteinuria. In the multivariable analysis, we identified an interaction between adiponectin and MIF levels, suggesting that high adiponectin levels combined with low concentrations of MIF is associated with the severity of proteinuria in LN. On the other hand, in the examination of renal dysfunction, we found that high MIF and resistin levels were correlated with an increase in serum creatinine in the entire SLE group. However, when we examined exclusively the subgroup of SLE with proteinuria, MIF and resistin concentrations did not remain correlated with serum creatinine, whereas resistin was also not correlated with eGFR when examined in the LN subgroup.

In this work, we evaluated proteinuria as the main surrogate variable of LN. Proteinuria is an important biological marker in LN, and proteinuria > 3.5 g/day is a risk factor for ESRD [20]. Patients with persistent proteinuria develop chronic renal disease with an increase in fibrosis in the renal tubules and renal interstitium [30]. MIF concentrations were related to a decrease in proteinuria in renal SLE, and this relationship remained in the multivariable analysis after adjusting for confounders. These findings are different from the previously published. Brown et al. identified an increase in urinary MIF that was observed only in proliferative nephritis, whereas no association was detected between serum or urinary MIF levels and the amount of proteinuria [17]. Vincent et al. found no associations between urinary MIF levels and LN, although elevated urinary MIF was observed in SLE patients with high disease activity [18]. These results suggest that the relation of MIF with the presence of LN is complex and requires an assessment of possible interactions with other molecules, including adipokines that could be associated with LN. MIF is a pleiotropic cytokine inducer of the synthesis of TNF-α and IL-6; additionally, MIF is a modulator of the inflammatory response, regulating T-cell proliferation [11]. In vivo studies have shown that MIF can abrogate the anti-inflammatory effect of glucocorticoids [31]. Studies in animal models demonstrate that blocking MIF induces a protective effect against inflammation in adjuvant-induced arthritis [32]. MIF can antagonise the immunosuppressive effect of glucocorticoids [33]. However, to date, it is not clear whether immunosuppressive treatments might modify MIF levels, whereas it has been suggested that TNF-α or IFN-γ might increase the release of MIF [34]. Instead, in our study no correlations were observed between MIF or leptin levels with glucocorticoids dose.

On the other hand, MIF concentrations correlated with an increase in serum creatinine only in the entire group of SLE patients but not in patients with renal activity. These data generate new questions regarding whether MIF could be a marker of renal dysfunction but not a marker of renal inflammation. Our findings are supported by the results observed by Otukesh et al., who identified a higher MIF/creatinine ratio in paediatric patients with LN than in those without LN [35].

In our study, high adiponectin levels were correlated with an increase in proteinuria. Nevertheless, many confounders can influence these results. Therefore, we performed a multivariable analysis, including those confounding variables that might affect the results. After this analysis, adiponectin levels remained a factor associated with proteinuria. Although we tested other adipokines in the present study, only adiponectin remained associated with the severity of proteinuria in SLE. These findings might reflect that adiponectin levels are a risk factor for proteinuria; nevertheless, these findings can also reflect an increase in adiponectin as a result of renal inflammation, with high serum levels acting as a potential protective homeostatic mechanism. An experimental study performed in cell cultures suggests that adiponectin can protect against the development of chronic renal disease by decreasing reactive oxygen species, local renal inflammation and fibrosis [36]. Another experimental study performed in cultured podocytes showed that the adiponectin regulation of inflammation could be mediated through the stimulation of AMP-activated protein kinase (AMPK) and a decrease in NADPH oxidase [37]. These experimental studies support our second hypothesis that adiponectin can have a role as anti-inflammatory molecule secreted in response to the stimuli of renal inflammation.

On the other hand, we observed a weak correlation between adiponectin and a decrease in eGFR, but we did not observe any correlation between adiponectin levels and an increase in serum creatinine. These findings are opposite to those observed by Hutchenson et al., who reported an association between adiponectin levels and renal dysfunction measured by serum creatinine [8]. However, our SLE patients had more disease activity than renal dysfunction, and the lack of a subgroup with very high levels of serum creatinine can influence the difference observed in our results.

Regarding the comparison between SLE and HCs groups. Our findings did not show differences in leptin between SLE and controls; instead, resistin was clearly increased in SLE patients compared with controls. Several publications have described increased levels of leptin and resistin in SLE patients [8, 38–41]. However, two different meta-analyses had opposite results regarding whether leptin levels are different in SLE vs controls [42, 43]. Regarding resistin, in a meta-analysis, Huang et al. did not identify differences between SLE patients and controls [44]. However, the heterogeneity of the characteristics of the SLE patients included in these meta-analyses can influence the variability of their results.

Finally, the findings of our study suggest that the relationship between adipokines and disease activity in SLE is more related with specific organs than a general increment of disease activity. We did not observe a correlation between the serum levels of leptin or resistin and disease activity measured by SLEDAI or MEX-SLEDAI. Our findings are supported by the results of the study performed by Santos et al., who did not identify an association of leptin or resistin with disease activity in SLE [45].

Regarding renal dysfunction and resistin levels, we found that serum resistin levels correlated with an increase in creatinine concentrations only in the entire group of SLE patients. Our findings were supported by the work performed by Hutcheson et al., who identified high resistin levels in SLE patients with renal dysfunction, including an increase in serum creatinine [8]. Nevertheless, we cannot conclude that resistin is a marker of renal dysfunction because in the subgroup of renal disease activity, the statistical significance of the correlations between resistin and serum creatinine or eGFR were lost.

To date, no previously published studies have evaluated the possible relation of serum adipokines with MIF levels in LN. Therefore, the present study was the first to assess a possible association of MIF and adipokines with proteinuria in SLE using a multivariable approach. Our findings support the interaction effect of serum levels of adiponectin and decreased MIF levels on proteinuria in SLE. The interaction that we observed between high adiponectin levels and lower MIF levels in patients with proteinuria secondary to LN might contribute to the identification of a different subgroup of patients with LN with more severe activity. However, it also allows us to formulate the question of whether our findings could reflect an adaptation mechanism involving increased adiponectin levels to limit renal damage in SLE nephritis and could be used to plan other therapeutic strategies.

There are some limitations in this work. First, the cross-sectional design represents only a snapshot of the complex relations of these inflammatory molecules with LN. Therefore, we ignored whether our findings of an increase in adiponectin together with a decrease in MIF levels might have occurred before the findings of renal relapse or if contrarily, adiponectin could have been increased after renal inflammation as an attempt to control the inflammatory process leading to low MIF levels. Follow-up studies are needed to solve this issue. Another limitation of our study was that the majority of our patients had a long SLE duration, and the characteristics of the cytokine and adipokine profiles might vary in patients with early SLE who develop nephritis. Additionally, a potential limitation of our study was that all of the SLE patients included were receiving treatment with glucocorticoids at the time of the study. We cannot exclude the effects associated with glucocorticoids on these molecules. An interesting observation published in the literature is that MIF is a cytokine that has been observed to decrease the effect of glucocorticoids [13]. Therefore, we ignored whether the MIF levels influenced the chain of events prior to the development of proteinuria in our SLE patients as a factor of corticosteroid resistance. Additionally, since glucocorticoids can increase the serum levels of some adipokines, further studies including a group of SLE patients prior to receiving glucocorticoids are needed to control for these confounders. Our results were derived from patients with SLE mainly with a long disease duration who were previously treated with glucocorticoids and immunosuppressive drugs, and a future study assessing LN patients with a recent diagnosis and measuring the molecules prior to starting immunosuppressive treatment is advised. Finally, a comparison of the serum levels of these analytes with urinary levels should be relevant to identify other associations. Future studies should take into account the importance of this comparison.

## Conclusions

In conclusion, with a multivariable approach, higher adiponectin serum levels combined with low concentrations of MIF were found to be associated with proteinuria in LN. After excluding the effect of other adipokines or MIF, this interaction remained. Leptin and resistin levels correlated with proteinuria. This study raises a new hypothesis that the levels of these molecules together could play an essential role as markers in LN. However, longitudinal studies including SLE of recent onset are needed.

## Data Availability

The datasets used and/or analysed during the current study are available from the corresponding author on reasonable request.

## Abbreviations

ACR: American College of Rheumatology
BMI: Body mass index
anti-dsDNA: Double-stranded DNA antibody
eGFR: Estimated glomerular filtration rate
HCs: Healthy controls
LN: Lupus nephritis
MIF: Macrophage migration inhibitory factor
MEX-SLEDAI: Mexican Systemic Lupus Erythematosus Disease Activity Index
rSLEDAI: Renal-SLEDAI
r_s_: Spearman rank correlation
SLE: Systemic lupus erythematosus
SLEDAI: Systemic Lupus Erythematosus Disease Activity Index
SLICC/ACR: Systemic Lupus International Collaborating Clinics/American College of Rheumatology
